# Health-related quality of life, physical and mental capacity at one year follow up of COVID-19 ICU patients: A prospective cohort study

**DOI:** 10.1186/s41687-025-00883-4

**Published:** 2025-05-14

**Authors:** Anders Ersson, Henrik Överengen Reuterborg, Anestis Divanoglou, Richard Levi, Lotti Orwelius

**Affiliations:** 1 Department of Anesthesia and Intensive Care, Nyköping Hospital, Nyköping, Sweden; 2 Faculty of Medicine, Department of Biomedical and Clinical Sciences, Linköping University, Linköping, Sweden; 3 Department of Rehabilitation Medicine and Department of Health, Medicine and Caring Sciences, Linköping University, Linköping, Sweden; 4 Department of Anesthesia and Intensive Care, Linköping University Hospital, Linköping, Sweden

**Keywords:** Critical care, Longitudinal analysis, Patient-reported outcomes, Quality of life, COVID-19

## Abstract

**Purpose:**

In 2020 as COVID-19 rapidly overwhelmed ICU resources, patient care capacity was reduced thus increasing the risk of development of post intensive care syndrome (PICS). Therefore, an increased incidence of survivors with neurocognitive and neuromuscular impairment could be anticipated. This study aimed to describe residual reductions in health-related quality of life (HRQoL) and risk factors for PICS as they pertain to outcomes one year after intensive care.

**Patients and Methods:**

Between 01-03-2020 and 31-08-2020, all adult COVID-19 ICU patients discharged alive in two Swedish ICU were included. At 2-, 6- and, 12-months post discharge follow up was conducted. Primary outcome parameters were HRQoL up to 12-months after ICU discharge. Secondary outcome parameters were clinimetric results for physical, mental, and cognitive functions at 6 months after intensive care stay.

**Results:**

Data from 41 patients were analyzed. Fatigue, anxiety, respiratory impairments, and experienced decline in physical stamina were the dominating findings at 6 months. Criteria for PICS were fulfilled in 93% of the study population and a 60% reduction in overall HRQoL, compared with a normal age adjusted population, was seen at follow up. A slight improvement was seen at 6 months whereafter no further significant improvement in HRQoL was detected. Fatigue was the most dominant complaint, expressed by almost all patients at follow up.

**Conclusion:**

Long term outcome reported in this study showed longstanding impairment in HRQoL, mostly related to reduced well-being and perceived limitations in physical ability. Overall, our findings show similarities with previously reported recovery patterns after intensive care. However, the COVID-19 cohort displayed a more profound reduction in HRQoL paralleled with severe fatigue and respiratory limitations. This signals the need for a deeper understanding of pathophysiological mechanisms of COVID-19 induced residual impairments and more precise instruments to tailor an individually designed aftercare.

**Supplementary Information:**

The online version contains supplementary material available at 10.1186/s41687-025-00883-4.

## Introduction

The advent of COVID-19 in 2020, immediately put a severe strain on global healthcare facilities and particularly on intensive care resources [1]. Beginning in March 2020, the situation rapidly deteriorated with a massive input of severely respiratory compromised patients that quickly overwhelmed ICU resources leading to patient care in a suboptimal ICU environment. Because of these circumstances, previously described risk factors [2, 3] for the development of post intensive care syndrome (PICS) such as ICU delirium, use of heavy sedation, especially with the use of Benzodiazepins, lack of spontaneous breathing trials and daily wake-up intervals and refraining from use neuromuscular blocking drugs were difficult to avoid. Thus, in comparison with outcomes typically seen after intensive care [4], a surviving cohort with an increased incidence of neurocognitive and neuromuscular impairments could be anticipated.

Post Intensive Care Syndrome (PICS) is a well described condition after critical illness [5, 6] comprising new or aggravated impairments with regards to physical (neuromuscular weakness and reduced independence in activities of daily living), mental (anxiety, depression, and post-traumatic stress disorder) and neurocognitive functioning, all of which negatively affect daily living and health related quality of life (HRQoL) [7, 8]. In over 50% of typical survivors after a period of intensive care, PICS manifests as a longstanding neuromuscular and cognitive compromise, resulting in decreased HRQoL up to several years after discharge [9]. The development of PICS and ICU delirium has been suggested to correlate with illness severity, ventilator treatment, the use of neuromuscular blockers and deep sedation [9] all of which were frequently present during the critical care of COVID 19 patients [2, 10]. As these patients also presented with severe pro-inflammatory laboratory profiles and had long ventilator times [10, 11], a prospect of major post intensive care complications could be expected.

Furthermore, COVID-19 induced respiratory failure, in which a substantially increased respiratory drive and impaired autonomous respiratory control made current recommendations of light sedation with daily wake-up and spontaneous breathing trials impossible or inappropriate. Thus, compliance with accepted guidelines for delirium prevention (ABCDEF- bundle) [12] were, in substantial parts, not manageable. Also, the acute shortage of nursing staff necessitated the need for simplified care routines, something which further contributed to a PICS promoting environment and amplification of predisposing factors.

During the first waves of the pandemic numerous authors highlighted the need for rehabilitation and often described the situation post- COVID as a novelty despite the apparent similarities to the situation post ICU generally seen [7, 13]. However, despite the fading of the pandemic phase, the advent of effective vaccination programs and a milder illness trajectory, the aftermath of this historic event necessitates the need to address persisting and long-standing post COVID sequels [13, 14] that are still affecting numerous patients.

A so called post- COVID Condition (PCC) [14] has been recognized and described as a conglomerate of symptoms, which, in large parts comprises symptoms analogous with the previously described post ICU residual infirmity (PICS). However, the picture is complicated by the fact that many patients have developed this syndrome in the absence of previous intensive care or even general inpatient care.

To date, the post- COVID patients displays longstanding limitations in their physical and cognitive capacities [9, 15–17] while the literature on long-term outcome and results of aftercare programs remains sparse. Although a need for rehabilitation is acknowledged, suggestions on how this recovery could be organized and constructed based on the data hitherto reported are still mostly lacking.

The aims of this study were twofold: first, to describe the clinical post-ICU trajectory and one-year development in health-related quality of life (HRQoL) in a cohort of ICU COVID-19 survivors from the first wave in 2020; and second, to identify risk factors for PICS-related impairments in neuromuscular and cognitive function, as well as their impact on HRQoL (Table 1).

**Table 1:** Presentation of clinimetric parameters

Category	Methods	Contents	Score range	Cut off value	Refs: [4, 10, 40–49]
Physical	Muscular strength and stamina	JAMAR, TST, 6MWT	<80% (age adjusted), >4% post exercise desat (6MWT)	–	Camarri
Respiratory function	Airway obstruction	PEF	–	<80% of expected	
	Spirometry	FEV1.0, VC, MIP, MEP	–	<80% of expected, results below gender and age adjusted expected values	Evans, Wahlgren
Cognitive	MoCA RBANS	8 Items 12 Items	0–30	<26 points (MoCA) 1.5 SD below the population-based mean (RBANS)	Nasreddine, Mikkelsen Randolph, Nakanishi, *Divanoglou*
Fatigue	MFI, FSS		20–100	<53, <5,2 score	Lundh, Valko Wahlgren
Anxiety and depression	IES, HADS	22 (IES), 14(HADS)	0–88 0–21	33 (IES) </=10(HADS)	Mikkelsen, Rousseau
HRQoL	Rand-36	36 Items	0–100	>10 score change	Kawakami, Rass, Orwelius

## Methods

### Design: Prospective multicenter cohort study

**Fig. 1:**
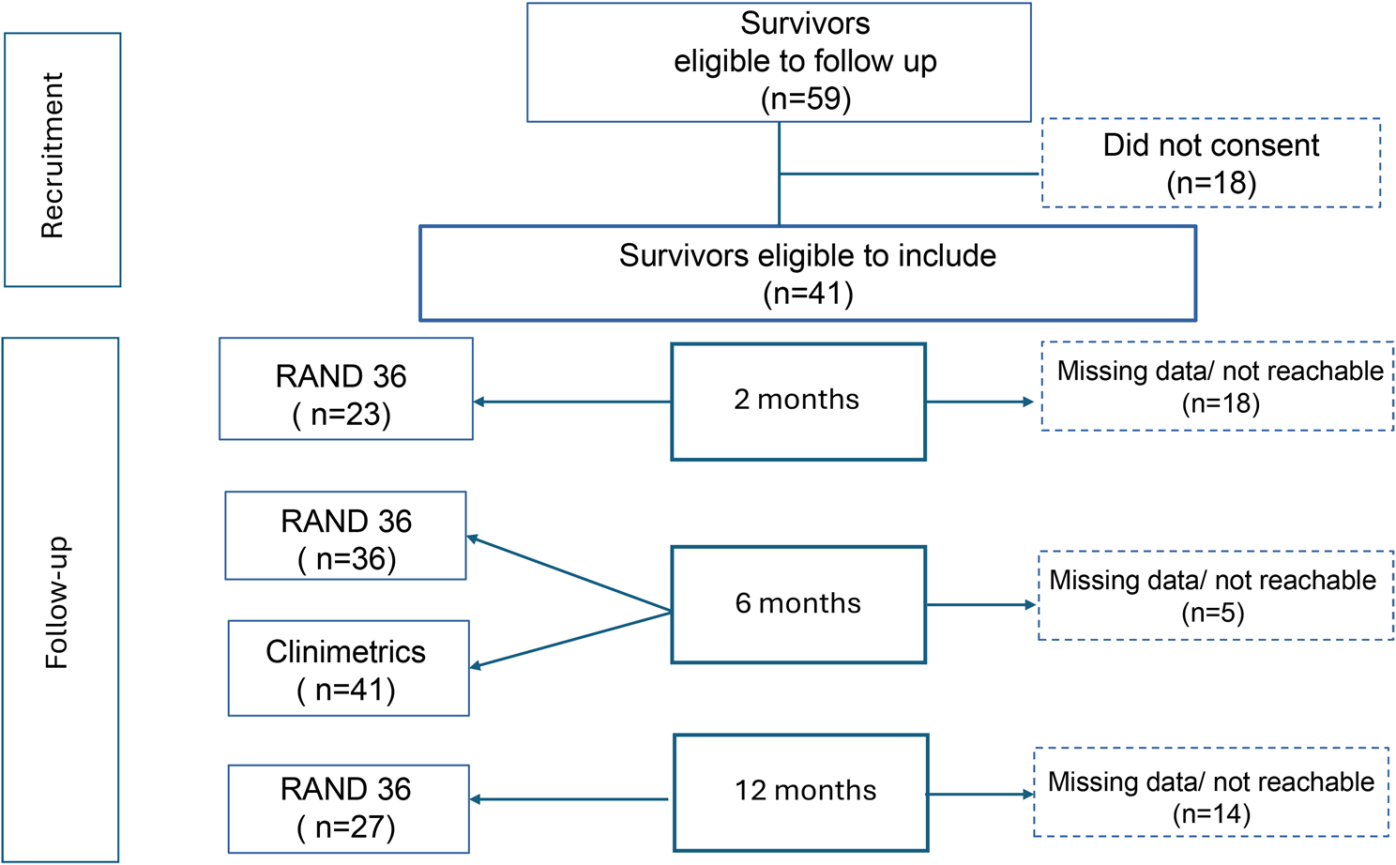
Study flowchart of participating patients

All adult (>18 years) COVID-19 patients with a length of stay >/=48 h and discharged alive from two ICUs between 01-03-2020 and 31-08-2020 were included. All survivors eligible to follow up were invited to participate and accepting patients were followed up regarding HRQoL 2-, 6- and 12-months post ICU discharge. Clinimetric testing (Fig. 1, Table 1) was performed at 6 months post discharge in both sites.

The data were collected in the ICUs post ICU clinics at the university hospital in Linköping (group 1) and at Nyköping hospital (group 2). Clinimetric data from Linköping were collected as part of the Linköping COVID-19 Study (LinCoS) conducted at the department of Rehabilitation Medicine, Linköping University Hospital.

The post ICU clinics in our hospitals were used to coordinate and provide aftercare services in collaboration with a multi -professional team consisting of paramedical services, rehabilitation medicine and ICU staff.

In our ICUs, all surviving patients with a length of stay of >/=48 h are routinely invited to attend a post -Intensive Care clinic at 2, 6, and 12 months after discharge. This is also the national Swedish standard for post ICU follow up suggested by a joint national expert panel [18].

At these clinics, the COVID-19 patients were evaluated by multidisciplinary teams (ICU and/or neurological rehabilitation) of physicians, nurses, physiotherapists, occupational therapists, social counsellors, and psychologists. Triage addressing the individual rehabilitation needs, trajectory and need for referral was conducted during the aftercare period.

Questionnaires for evaluation of HRQoL, anxiety and depression, sleep disorders and fatigue were mailed to all participating patients to fill in before attending the post ICU clinic. During the visit these forms were evaluated, and the patients also underwent clinimetric testing to evaluate physical capacity, pulmonary function, cognitive capacity, and ADL functions.

PICS is defined as new or worsening impairment in *Physical capacity* (ICU-acquired neuromuscular weakness), *Cognitive capacity* (thinking and judgment), or *Mental Health status* arising after critical illness and persisting beyond discharge from the acute care setting [19]. PICS were considered present if the patient presented with values below the described thresholds in the qualities: *Mental Heath status* (HADS, IES-R), *Cognitive capacity* (MOCA, RBANS) and/ or *Physical capacity* tests as described below.

At the face-to-face follow up, the stay in the ICU was recapitulated and the patient was given a diary written during their stay in the ICU.

### Clinical variables

#### Patient characteristics

Age, sex, Body Mass Index (BMI), Charlson Comorbidity Index (CACI), illness severity, Simplified Acute Physiology Score (SAPS III), Clinical Frailty Score (CFS), Time on ventilator, hospital and ICU length of stay (LOS) were analyzed.

### Health related quality of life

The public domain form (RAND-36 Item Health Survey) questionnaire [20] was used to assess health related quality of life (HRQoL). This questionnaire was developed in the RAND Medical Outcomes Study during the 1980s [21]. Two identical versions of the questionnaire are available: the RAND-36, and the copyrighted, commercially distributed form SF-36 Item Health Survey [22]. The instruments consist of 36 items divided in eight dimensions, exploring physical and mental aspects of daily living; Physical function (PF), Physical role (RP), Bodily pain (BP), General health (GH), Vitality (VT), Social function (SF), Emotional role (RE), and Mental health (MH). The scores on all dimensions are transformed to a scale from 0 (the worst score) to 100 (best score). Normal values for a general Swedish population have been previously described [4] and used as reference.

A difference of >5 units from reference values were considered pathological and is claimed as a clinically significant effect change between timepoints [23].

### Clinimetrics

The results from the different tests were categorized as normal/pathological using previously suggested cut of values described in the literature (Table 1).

#### Cognitive function

Cognitive function was tested using either the Repeatable Battery for the Assessment of Neuropsychological Status (RBANS), (group 1) or Montreal Cognitive Assessment test (MoCA), (group 2). Significant neurocognitive impairment was defined as 1.5 SD below the population-based mean. (RBANS). A result <26 points (MoCA), was considered pathological.

### Anxiety and depression

Hospital Anxiety and Depression Scale (HADS) and Impact of Events Scale (IES-R) were used for evaluation. Thresholds for pathological results were >/=10 (HADS) and >/=33 points (IES-R).

### Physical function

This was evaluated using handgrip strength (JAMAR), Time Stand Test (TST) and Six Minutes Walk Test (6MWT). A result <80% of normal adjusted for age was considered pathological. The 6MWT was also evaluated in terms of desaturation (D) and short walking distance (SD) which was adjusted for age. A post exercise desaturation >4 % of resting value was considered pathological.

### Respiratory function

Respiratory capacity was evaluated using (Peak Expiratory Flow PEF, group 2), Spirometry (group 1) and Maximal Inspiratory/Expiratory Pressure (MIP, MEP, group 2). For PEF <80% of an age adjusted normal value was considered pathological.

Normal means that FEV1, PEF and VC are all at least 80% of their respective expected values. Pathological indicates that at least one of: FEV1, PEF and VC are below 80% of the expected value and (b) no known respiratory co-morbidity exists.

For MIP and MEP results below gender adjusted lower limits of normal were considered pathological.

### Fatigue

Multimodal Fatigue Inventory (MFI) (group 1) and Fatigue Severity Score (FSS) (group 2) were used. MFI and FSS scores below 53 and 5.2 respectively were considered pathological.

### Ethical considerations

This study was conducted in accordance with the declaration of Helsinki and with Ethical permission from The Swedish Ethical Review Authority (Dnr. 2020-03029, 2020–07121 and 2020-04012).

All patients and relatives were informed that clinical and follow-up outcome data were collected as part of national routine quality assessment. All participants were informed that they could withdraw from the study at any time and that their data would then not be analyzed and presented on an individual basis.

### Statistical analysis

Demographic data for each hospital are presented as median and inter quartile range (IQR). Differences between groups were analyzed using Chi-Square for nominal data, parametric (independent *t*-Test) and non-parametric tests (Mann Whitney) where normal distribution of the sample were considered non-existent (HRQoL data). For repeated measures over time Friedman two-way analysis of variance were used.

A *p* value of <0.05 were considered significant.

Primary outcome parameter: HRQoL at 2-, 6- and 12-months post ICU.

Secondary outcome parameters: Clinimetric results for physical and cognitive functions at 6 months after intensive care.

Demographic and clinimetric variables (BMI, CFS, SAPS3, Time on ventilator, Comorbidity, HAD-Angst, HAD-Depression, MOCA, RBANS, and MFI/FSS) were subjected to univariate linear regression, adjusted for age and sex. A *p*-value </=0.05 was considered significant. Variables that were significant on univariate analysis (Time on ventilator, Comorbidity, HAD-Angst, HAD-Depression, and MFI/FSS) were subjected to multivariate linear regression analysis, adjusted for age, and sex, for identifying risk factors for impaired HRQoL (see Supplemental material). In these models all variables were introduced as continuous variables

## Results

A total of 41 patients were finally included in the analysis (Fig. 1).

Fatigue, Anxiety, respiratory limitations and an experienced decline in physical stamina dominated the situation post ICU at 6 months.

**Fig. 2:**
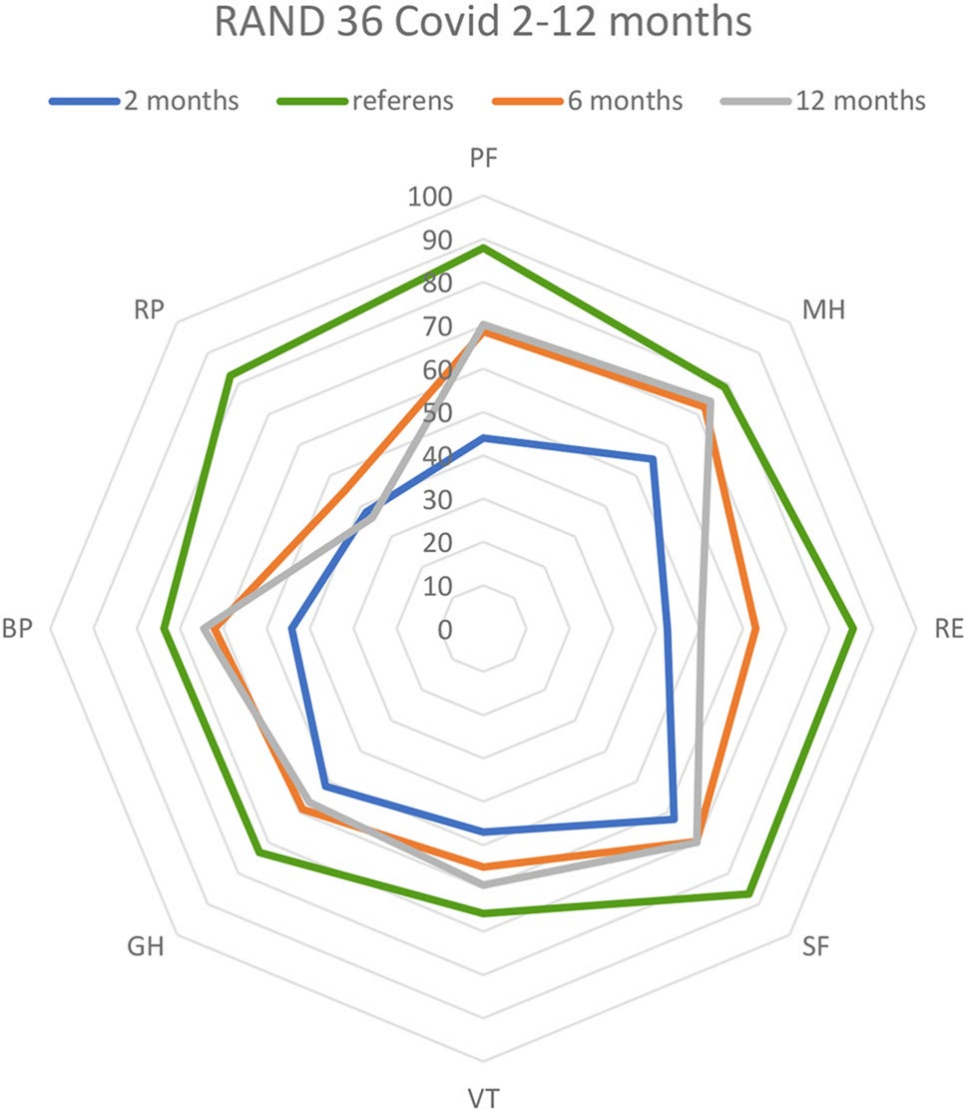
Development in RAND-36 during the observation period. The reference group consists of a normal age-adjusted healthy Swedish population. PF: Physical function, MH: Mental health, RE: Role emotional, SF: Social function, VT: Vitality, GH General health, BP: Body pain, RP: Role physical

Most patients (93%) fulfilled the criteria for PICS as defined above and experienced a substantial impairment in HRQoL with low scores in physical, mental, and cognitive capacity domains at 2 months. The scores improved slightly at 6 months post ICU except for persisting low values in the dimensions RP and RE. There were no further improvements in HRQoL from 6 to 12 months post ICU (Fig. 2).

### Demographics

The median age was 60 years of age, and the group included 73% males. The median LOS in the ICU was 15 days and total hospital stay was 29 days (Table 2a).

**Table 2a:** Patient characteristics-cohort

Characteristics of study participants n = 41	Median	IQR
Age (y)	60 (54–68)	14
Gender male %	72.9	
Height (cm)	172 (163.8–180)	16.3
Weight (kg)	82 (25.8–101.5)	27.7
BMI	27.9 (25.8–33.1)	7.3
CACI	3 (1–4)	3
CFS	3 (2,3)	1
SAPS-3	54 (49.8–60)	10.3
IMV (%)	92	
IMV (h)	336 (172–578)	406
LOS ICU (d)	15 (8.4–24.4)	16
LOS Hospital (d)	29 (13–46.4)	33.4

Patient characteristics for the pooled cohort

Data is given as Median (Q1–Q3) and IQR unless otherwise shown

BMI (Body Mass Index); CACI (Charlson Comorbidity Index); CFS (Clinical Frailty Scale); SAPS-3 (Simplified Acute Physiology Score); IMV (Invasive mechanical ventilation); LOS (Length of Stay)

The population was slightly overweight with a BMI of 27.9. Despite a high SAPS score of 54, the group showed a comparatively low rate of frailty and co-morbidity displayed as a low median clinical frailty and CACI of 3, indicating that only minor co-morbidities and impairments in daily activities were present prior to the ICU period. No significant differences were seen in patients’ characteristics between the groups, except for a trend towards a slightly higher BMI in group 2 with group mean of 30 vs 27 (*p* = 0.02 at *t*-test) but identical medians of 28 (Table 2b). In addition, there was a slight difference in co- morbidity, CACI (*p* = 0.04) with group 1 reporting a group mean of 3 vs 2 in group 2 Despite these minor differences in patient characteristics, the decision was taken to pool the group results in the subsequent analysis of clinimetric and HRQoL data (Table 2b).

**Table 2b:** Patient characteristics-groups

Characteristics of study participants	Median Group 1	IQR Group 1	Median Group 2	IQR Group 2	p
Age (y)	63	14.3	59	14	0.32^a^
Gender male %	79		69		0,37^b^
BMI	27.6	4	2.4	7.9	0.02^a^
CACI	3	2	2	2	0.04^b^
CFS	3	1	3	1	0.64^b^
SAPS-3	53.5	7.8	54	12	0.47^a^
IMV (h)	335.8	354.8	336	492	0.26^a^
LOS ICU (d)	15	13	15	19	0.51^a^
LOS Hospital (d)	27	33	31	33.5	0.64^a^

Patient characteristics groupwise and comparison between the two groups

Data is given as Median (Q1–Q3) and IQR unless otherwise shown

BMI (Body Mass Index); CACI (Charlson Comorbidity Index); CFS (Clinical Frailty Scale); SAPS-3 (Simplified Acute Physiology Score); IMV (Invasive mechanical ventilation); LOS (Length of Stay)

^a^Analyses method: *T*-test

^b^Analyses method: Chi-Square

### HRQoL

At 2 months post-ICU, all HRQoL dimensions were lower compared to an age-adjusted reference population [4]. The most severe deviations were observed in the dimensions of PF, RP, and RE (Fig. 2). Within the study group, there was an improvement in all dimensions at 6 months whereafter no significant improvement was detected (Table 3). However, for RP and RE, the results remained low and even deteriorated slightly at 12 months. The improvement at 6 months was most dominant in PF, BP, SF, and MH but did not show further progress or returned to normal during the study period (Table 3, Fig. 2).

**Table 3:**
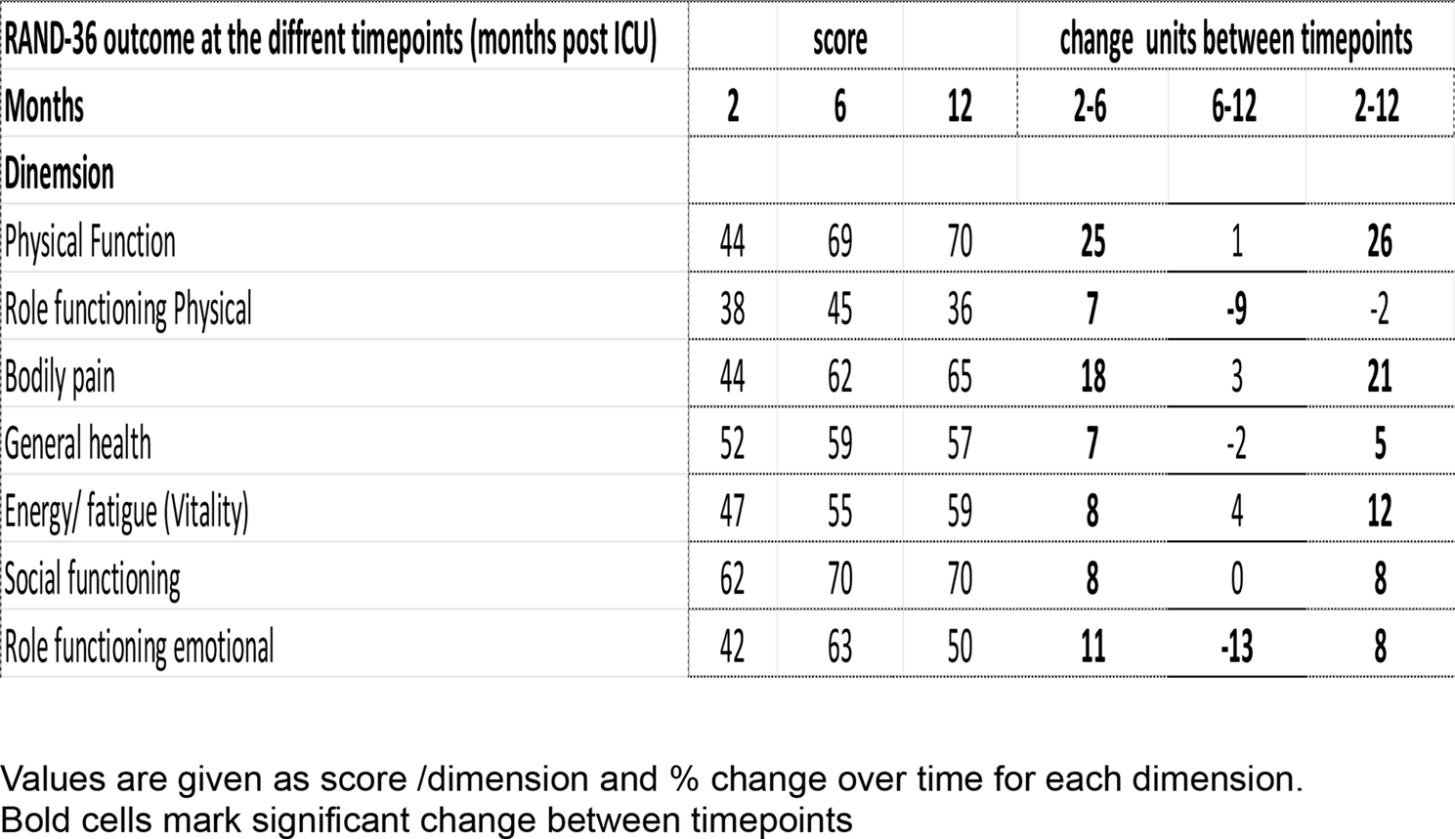
Health related quality of life at the different timepoints

There was a reduction in the experienced physical and emotional capacity, which showed a constant decline during the study period. This was expressed as persistent low scores in the RP and RE dimensions (Table 3, Fig. 2)

### Clinimetrics

See Table 4.

**Table 4:** Results of the clinimetric testing

Category	Item	% pathological
Cognition		
	HADS total score	50
	HADS anxiety	22
	HADS depression	11
	IES	55
	MOCA	47
	RBANS	24
Muscular function		
	TST	44
	JAMAR	38
	6MWT	86
		(D) 20
		(SD) 80
Fatigue		
	FSS	100
	MFI	94
Lung function		
	Spirometry, MIP/MEP	43
	PEF	0

Results of clinimetric measurements

#### Cognitive function

Clinimetric follow up was performed at 6 months in both groups. At 6 months there was a profound impairment in cognitive performance with 47% (MOCA) and 24% (RBANS) of the cohort showing cognitive results below threshold.

#### Neuro- psychiatric parameters

Anxiety and depression were frequently seen in the patients and were a dominating factor in the overall reduction in HRQoL, which is described below in the univariate and multivariate analyses. Fifty percent of the patients scored pathologically in the HAD display in which anxiety was the dominating quality. Likewise, the patients expressed a high frequency of disturbing memories and experiences with 55% displaying high scores measured by IES-R.

### Physical capacity

Physical performance was generally impaired. Muscular strength was reduced in 38% (handgrip) and in 44% (standing) of the population and 86% showed limited walking distance while only 20% displayed a post exercise desaturation.

#### Lung function

Shortness of breath was frequently reported by the patients at the post ICU clinic and was a persistent complaint throughout the follow-up period. This was often described as limiting in daily activities and 43% had objective reductions in lung function either as results below normal age adjusted values in dynamic spirometry or in MIP/MEP readings. The reduction in respiratory capacity did not seem to be of an obstructive nature as all patients tested displayed a normal PEF.

### Fatigue

Fatigue was the most dominant and persisting complaint by the patients and was often described as severe and disabling with a profound impact on the ability to carry out daily activities and cognitive tasks. Virtually all patients in the cohort scored pathologically in this quality (100% and 94% respectively in the two groups).

### Univariate regression analysis

The univariate analysis showed significant influences on various dimensions in RAND-36. Of these parameters HAD and fatigue significantly influenced all dimensions. Time on ventilator and comorbidity were significantly correlated with impaired score in the RP, RE, GH and VT dimensions (supplemental material).

### Multivariate regression analysis

Parameters that showed significance in the univariate analysis underwent further testing. In the multivariate analysis, only HAD showed a significant detrimental influence in the dimensions MH and SF and co-morbidity in RP (supplemental material).

## Discussion

The long-term outcomes reported in this study showed profound impairments in HRQoL, mostly related to reduced mental well-being and perceived limitations in physical ability.

The post-COVID situation has, as expected, evolved into a serious health problem. Many former COVID-19 patients continue to experience physical and cognitive limitations and reduced HRQoL, even years after the advent of the pandemic despite ambitious follow up programs, Post COVID clinics, increased clinical knowledge and experience [15, 16]. Fatigue, shortness of breath, PTSD, anxiety/depression, and cognitive problems are frequently reported in post-ICU patients [24], with these symptoms also dominating our study. Fatigue also seems to be a dominating feature of the Post Covid syndrome, even in patients not treated in ICUs. In the univariate analysis, fatigue significantly influenced all dimensions in HRQoL except physical (RP). However, it was not identified as a significant component for HRQoL outcome in the subsequent multivariate analysis. The role of fatigue in the composite trauma resulting from a period of intensive care for COVID-19 remains unclear and might be a secondary effect to anxiety and depression frequently expressed by these patients.

While the recovery patterns of our study group resembled what is typically seen after intensive care, we believe that the ICU-treated COVID-19 cohort differs in certain aspects. Co-morbidity and frailty have earlier been identified as strong predictors for reduced HRQoL after intensive care [4, 11, 25, 26]. However, the assessment of these factors varies across studies and, in the ICU setting, tools like SAPS, CACI and CFI may not fully capture the impact of co-morbidities on long-term HRQoL outcomes.

In our material, co-morbidity, measured using the CACI, showed some association with HRQoL. Specifically, CACI had a significant influence on perceived physical performance (RP), a dimension that often remains low over time after intensive care.

At 2 months, the COVID cohort showed a more profound overall reduction in RAND-36 compared to a normal ICU cohort [27]. However, CFI and CACI scores remained at moderate levels. This is notable, given that both CACI and CFI are based on predefined conditions primarily associated with mortality risk [28] and may not adequately reflect the broader range of impairments relevant to HRQoL.

These findings highlight the need for better markers of comorbidity that are sensitive to pre-existing conditions and relevant to the unique context of post-COVID ICU survivors. Tools like CACI and CFI may be insufficient for identifying impairments in HRQoL in this population. Here, a complementary patient-reported experience measure (PREM), specifically designed to capture the ICU experience, could help better identify the mental and cognitive impairments that influence post-ICU outcomes, as described in our study.

Severe fatigue and respiratory limitations, possibly leading to incapacitating and persistent reductions in HRQoL stood out in our study and might be some of the main drivers behind the reduction in overall HRQoL. Fatigue has earlier been described in COVID-19 survivors [5, 15, 16, 24, 29] and seems to differ in intensity from a normal cohort of ICU survivors [30, 31] Also, presence of fatigue symptoms at 6 and 12 months after intensive care has been associated with worsening of physical functioning and psychological impairments [9, 32] which was confirmed in our results.

In our study we also saw the co-occurrence of cognitive impairment and fatigue, a pattern previously described in the COVID-19 cohorts [29]. Although physical limitations have been shown to co-variate with mental impairments, a clear association with cognitive impairments has not been established [9]. Our results revealed a discrepancy between patients’ perceived limitations in physical function, reflected in persistently low RP scores (RAND-36) and the results from the physiological tests. Fatigue, anxiety and depression were dominating the clinimetric assessments with >50% of patients showing pathological values along with low MoCA scores (Table 4). Based on the above, we suggest that patients’ perception of their physical abilities may have been negatively influenced by the mental and cognitive state post ICU. This could have had a detrimental influence on the initial self -assessment, as well as on the long-term recovery process. The initial improvement observed at 6 months in all RAND-36 dimensions, except RP, likely reflects recovery in severely compromised somatic functions, particularly those related to neuromuscular and respiratory compromise developed during ICU stay. The lack of further progress beyond 6 months aligns with patterns seen in general critical care patients. However, as discussed, persistent mental and cognitive impairments likely contributed to this stagnation.

The finding of severe fatigue and breathlessness in our study is striking compared to our previous experience of normal ICU recovery, but well in line with previously reported results [24]. This might be instrumental in causing the initially much lower overall scoring at 2 months and the persistently low scoring in RAND-36 in the following timepoints. Apart from that, the general trajectory in HRQoL did not seem to differ considerably at 6 months from the post ICU HRQoL situation in a regular ICU cohort [27] with the exceptions of the dimensions MH, RE, and RP. This is perhaps to be suspected, considering the circumstances under which the ICU care for patients with COVID-19 was provided and that the dimension MH in RAND-36 reflects the high scores in anxiety and depression.

As mentioned, fatigue, in our study and in those of others [5, 15, 16, 24, 29] stands out as exceptional in the COVID-19 cohort and may be pivotal for the impairments seen in HRQoL as well in cognitive and physical performance. Thus, this merits a discussion on possible mechanisms, as it largely determines the patient´s potential for recovery and therefore must be addressed individually when tailoring aftercare, rehabilitation, and appropriate interventions.

Activation of inflammatory and coagulation pathways are a paramount feature in sepsis and ARDS and because they are associated with the development of multiple organ failure, they are also suggested to propel PICS development and to promote dementia and disability [32]. Considering the massive activation of inflammatory and coagulation pathways experienced during the COVID-19 pandemic one might suspect an inflammatory culprit as a plausible catalyst of residual symptoms and, in particular, for making fatigue a dominating component in the post -COVID syndrome. This seems plausible considering earlier studies of patients after intensive care that demonstrated profound cognitive deficits and loss of brain tissue, particularly in white matter, on ability testing and neuroimaging [8, 30, 34]. The neuronal damage visualized on neuroimaging may have a correlate in an altered regulation of micro and astroglia as earlier demonstrated in traumatic brain injury and post viral fatigue [34–36].

Although there is no evidence of a direct viral invasion of brain tissue, reactive micro and astroglia cells together with inflammatory infiltrates can be seen in 44–52% of COVID-19 patients at autopsy and as such represent the effects of a generalized inflammation penetrating the blood brain barrier [30, 36].

However, a correlation between long-term cognitive outcomes and inflammation has not yet been fully established and shows inconsistent associations with disability outcomes [37].

In our study mental and cognitive residual symptoms in companion with shortness of breath dominated the post-COVID picture. In the light of the massive inflammatory activation created by the COVID-19 infection it is plausible that the inflammatory response could be a pivotal factor that could be pivotal for the severe fatigue and cognitive impairments experienced by the patients. This, together with nursing in an extremely stress-filled environment in which normal PICS preventive routines were forced to be abandoned might have contributed to the high amount of depression and anxiety reported. Taking together, these circumstances could explain the persistently poor results in HRQoL, especially in the dimensions concerning experienced physical function and mental wellbeing.

It should be noted that post COVID syndrome occurs also, not uncommonly, in milder cases of infection, where neither ICU admission nor even hospitalization was needed. However, displaying similar symptoms, cognitive impairments, PTSD, pain, and anxiety have been reported to dominate among ICU patients. Fatigue and breathlessness were commonly seen in both groups but again, they were more prominent in the ICU group [38]. Thus, when assessing persisting symptoms after ICU-treated COVID-19, it seems highly unlikely to attribute the syndrome exclusively to ICU-related factors. Rather, it is more plausible to hypothesize that illness severity and the infection as such are the main drivers, with ICU-related factors adding to the insult.

Even though the symptomatology of the post COVID syndrome has been described and the patient’s situation have been addressed previously, the pathophysiology is unclear still and demands further studies concerning inflammatory neurotrauma and possible preventive measures. In addition, and as previously addressed [39], in our perspective, tailoring these efforts in accordance with clinical findings and results obtained during the aftercare process is crucial for optimizing individual recovery trajectories in accordance with specific needs of each patient.

## Strengths and limitations

In this study, we used only validated instruments when investigating areas known to be influenced after intensive care (HRQoL, anxiety and depression, fatigue, and neurocognitive impairments). Furthermore, it is notable that the patients were followed up on more than one occasion, which is essential as it captures important changes over time. A further strength is that the cohort from the two sites included in the study were, by and large, homogenous in their characteristics, which is unusual even for a single ICU cohort.

Some limitations in this study were identified. First, we had no control group data analyzed for HRQoL during the same period as the study group. Instead, we used data from a general reference group collected several years before our study [4]. This could have skewed the comparisons as the health in the community may have changed over time. Therefore, when evaluating RAND-36 we also used open data from the Swedish Intensive care register as a contemporary reference. Another limitation is that we pooled two sites with different organizations and sizes comprising one university hospital and one county hospital. However, as there were only minor differences between the two cohorts in patient characteristics, we argue that this should have an acceptable and minor impact on the results. Further, we were limited to using clinimetric data from only the 6 months clinic as the clinimetric follow up was performed on all three follow up occasions in one center only. However, since no improvement in HRQoL was seen after 6 months one can argue that the clinimetric situation at 2 months well represents the level of recovery achieved in the cohort over time. The use of different clinimetric instruments at the different sites was not optimal but results from the different routines and instrument inventories normally used for follow-up at the two sites. Considering the overwhelming clinical workload during the height of the pandemic when data was collected, we decided that it was more important to capture patient data for follow-up than to simultaneously try to change and implement new routines. Because of this, we only report the results binarily as pathological/non pathological and not in absolute numbers.

A consideration is also whether there was a selection bias in the data concerning patients lost to follow-up or who did not respond at all (Fig. 1). Apart from several patients not having Swedish as their native language, we cannot exclude that many patients either recovered and thus lacked motivation to respond, or alternatively that patients with cognitive impairment due to their disability did not answer the questionnaires. In our analysis on dropouts and missing data we see that despite a similar illness severity, the non- responders were predominantly male and had longer time on ventilator and longer ICU stay than the responders (Table 5). This might indicate that these patients were more likely to have more pronounced sequelae and thus lesser ability to participate in the follow- up clinics or answer the questionnaires. Either way, this would have the potential to influence scoring in HRQoL after critical illness.

**Table 5:** Comparison of patients lost to follow-up to the responders

Group	Age	Male %	CFS	SAPS III	Vent time (h)	LOS ICU (d)
Responders (*n* = 41)						
mean	60		3	54	420	18
		60				
Non responders (*n* = 18)						
mean	56		3	56	519	20
		89				
*p*-value (significant values in bold format)	0.749		0.222	**0.006**	**0.001**	**0.012**

Lastly, the use of CFI and CACI for evaluating pre-existing conditions and co-morbidity may not be appropriate in a setting focused on HRQoL since these instruments mainly correlate with mortality.

## Conclusion

This study shows that, compared with an age-adjusted reference population, the patients cared for who had COVID-19 perceived a reduction of physical and emotional capacity with a decline persisting at 12 months after discharge from the ICU. Fatigue was the most dominant complaint and was seen in almost all patients in the study cohort. These findings underline the importance of accounting for fatigue and for the exploration of possible pathophysiological mechanisms when tailoring the aftercare, rehabilitation, and appropriate interventions after intensive care.

## Electronic supplementary material

Below is the link to the electronic supplementary material.

Supplementary Material 1

Supplementary Material 2

## Data Availability

All study raw data, such as patients ‘records, test forms, post ICU clinics protocols and, CRF are stored in locked cabinets at the respective hospitals and is not available to the public with respect to patient secrecy. The anonymized datasets and statistical data sets used for the analysis are stored on separate hard drives by the research group. The datasets used and/or analyzed during the current study are available from the corresponding author on reasonable request.

## Abbreviations

PICS: Post intensive care syndrome
HRQol: Health related quality of life
ICU: Intensive care unit
PCC: Post covid condition
LOS: Length of stay
ADL: Activities of daily living
HADS: Hospital anxiety and depression scale
IES-R: Impact of events scale-revised
MoCA: Montreal cognitive assessment test
RBANS: Repeatable Battery for the Assessment of Neuropsychological Status
BMI: Body mass index
CACI: Charlson comorbidity index
SAPS: Simpified acute physiology score
CFS: Clinical frailty score
PF: Physical function
RP: Physical role
BP: Body pain
GH: General health
VT: Vitality
SF: Social function
RE: Emotional role
MH: Mental health
TST: Time stand test
6MWT: Six minutes walk test
PEF: Peak expiratory flow
MIP/MEP: Maximal inspiratory/expiratory pressure
FEV1.0: Forced expiratory volume during 1second
VC: Vital capacity
MFI: Multimodal fatigue inventory
FSS: Fatigue severity score

## Declarations

### Anders Ersson. MD, PhD, DEAA, EDIC, Associate professor

Main author and responsible for manuscript preparation, data analysis and, editing of the final manuscript. Primary investigator at Nyköping hospital.

### Henrik Överengen-Reuterborg. MD, Resident in Anesthesia and Intensive care

Responsible for development and monitoring of study protocols, data collection and co- authorship during the manuscript preparation.

### Anestis Divanoglou. Physiotherapist, PhD

Responsible for data collection and processing of study protocols at Linköping University hospital. Collaboration in the data processing and statistical analysis of the material and in editing of the manuscript during the preparation process.

### Richard Levi. Professor

Collaboration in editing of the manuscript during the preparation process and in the collection and compilation of patient data.

### Lotti Qrwelius. RN, CCN, PhD, Associate professor

Coauthor in the manuscript preparation process and editing. Responsible for the statistical analysis and processing of the material. Primary investigator at Linköping University hospital.
