# Supplementary material for: Health-related quality of life, physical and mental capacity at one year follow up of COVID-19 ICU patients: A prospective cohort study
Source: J Patient Rep Outcomes. 2025 May 14;9:52. doi: 10.1186/s41687-025-00883-4 (PMC12078742; doi:10.1186/s41687-025-00883-4)
Supplement: Supplementary file 1 — Supplementary Material 1 [file 41687_2025_883_MOESM1_ESM.docx]

**Multivariate linear regression for investigating influence on HRQoL at six months post ICU discharge**

|  |  |  |  | Dimensions | |  |  |  |  |
| --- | --- | --- | --- | --- | --- | --- | --- | --- | --- |
| Variable | **PF**  P Value  Beta coeff /  R^2^ | **RP**  P Value  Beta coeff /  R^2^ | **BP**  P Value  Beta coeff /  R^2^ | | **GH**  P Value Beta coeff /  R^2^ | **VT**  P Value  Beta coeff /  R^2^ | **SF**  P Value  Beta coeff /  R^2^ | **RE**  P Value Beta coeff /  R^2^ | **MH**  P Value  Beta  coeff /  R^2^ |
| **Time on ventilator** | 0.42  -0.02/  0.355 | 0.442  -0.02/  0.531 | 0.197  -0.02/  0.599 | | 0.160  -0.12/  0.518 | 0.440  -0.01/  0.694 | 0.475  -0.01/  0.608 | 0.400  -0.31/  0.367 | 0.187  -0.01/  0.854 |
| **Comorbidity** | 0.323  16.6/  0.355 | **0.033**  56.87/  0.531 | 0.775  4.15/  0.599 | | 0.244  12.32/  0.518 | 0.157  17.49/  0.694 | 0.872  2.42/  0.608 | 0.662  13.14/  0.367 | 0.667  -3.03/  0.854 |
| **HAD Angst** | 0.573  0.97/  0.355 | 0.180  3.53/  0.531 | 0.259  -1.61/  0.599 | | 0.896  -0.14/  0.518 | 0.914  -0.13/  0.694 | 0.565  -0.90/  0.608 | 0.491  -2.16/  0.367 | **0.002**  -2.65/  0.854 |
| **HAD Depr.** | 0.371  -1.70/  0.355 | 0.084  -5.09/  0.531 | 0.117  -2.69/  0.599 | | 0.182  -1.62/  0.518 | 0.259  -1.57/  0.694 | **0.03**  -4.03/  0.608 | 0.126  -5.44/  0.367 | **0.001**  -3.25/  0.854 |
| **MFI/ FSS** | 0.781  -0.111/  0.355 | 0.559  -0.35/  0.531 | 0.694  -0.14/  0.599 | | 0.864  -0.04/  0.518 | 0.187  -0.39/  0.694 | 0.593  -0.20/  0.608 | 0.480  0.52/  0.367 | 0.472  0.14/  0.854 |

Data are analyzed at 95% CI. A p value </=0.05 is considered significant - Boldface indicates significant p-value. Data is adjusted for age and sex.
