# Supplementary material for: Health-related quality of life, physical and mental capacity at one year follow up of COVID-19 ICU patients: A prospective cohort study
Source: J Patient Rep Outcomes. 2025 May 14;9:52. doi: 10.1186/s41687-025-00883-4 (PMC12078742; doi:10.1186/s41687-025-00883-4)
Supplement: Supplementary file 2 — Supplementary Material 2 [file 41687_2025_883_MOESM2_ESM.docx]

**Univariate linear regression for investigating HRQoL at six months post ICU discharge**

Supplemental material

|  |  |  |  | Dimensions | |  |  |  |  |
| --- | --- | --- | --- | --- | --- | --- | --- | --- | --- |
| Dep.  Variable  Factors | **PF**  P value  Beta-coeff/  R^2^ | **RP**  P value  Beta-coeff/  R^2^ | **BP**  P value  Beta-coeff/  R^2^ | | **GH**  P value  Beta-coeff/  R^2^ | **VT**  P value  Beta-coeff/  R^2^ | **SF**  P value  Beta-coeff/  R^2^ | **RE**  P value Beta-coeff/  R^2^ | **MH**  P value  Beta-coeff/  R^2^ |
| **BMI** | 0.061  1.56/0.097 | 0,212  1.54/0.079 | 0.206  1.12/0.083 | | 0.992  0.01/0.085 | 0.74  0.25/0.025 | 0.447  0.69/0.082 | 0.905  0.16/0.004 | 0.441  0.52/0.054 |
| **CFS** | 0.979  0.16/0.006 | 0.566  5.09/0.047 | 0.786  1.74/0.044 | | 0.719  1.57/0.089 | 0.901  0.65/0.023 | 0.381  5.64/0.087 | 0.126  14.82/0.065 | 0.395  4.06/0.058 |
| **SAPS 3** | 0.972  0.02/0.006 | 0.801  0.24/0.04 | 0.905  0.08/0.043 | | 0.337  0.45/0.108 | 0.596  0.30/0.03 | 0.783  0.19/0.07 | 0.653  0.48/0,009 | 0.269  0.54/0.067 |
| **Time on ventilator** | 0.099  -0.03/0.16 | **0.035**  -0.06/0.2 | 0.065  -0.04/0.18 | | **0.019**  -0.03/0.41 | 0.15  -0.03/0.19 | 0.284  -0.02/0.13 | **0.009**  -0.06/0.10 | 0.188  -0.02/0.17 |
| **Comorbidity** | 0.109  16.55/0.14 | **0.01**  42.766/0.28 | 0.147  16.81/0.15 | | **0.042**  15.17/0.34 | **0.043**  20.07/0.24 | 0.273  24.76/0.13 | 0.589  9.88/0.01 | 0.773  2.53/0.15 |
| **HAD Angst** | 0.408  -1.22/0.06 | 0.9  -0.29/0.09 | **0.01**  -3.63/0.32 | | 0.092  -1.56/0.26 | **0.051**  -2.55/0.30 | **0.01**  -3.69/0.33 | 0.073  -4.37/0.15 | **<0.001**  -3.88/0.59 |
| **Had Depr.** | 0.096  -2.71/0.15 | 0.077  -4.46/0.22 | **<0.002**  -4.812/0.42 | | **0.014**  -2.48/0.37 | **0.006**  -3.92/0.42 | **<0.001**  -5.643/0.54 | **0.014**  -6.52/0.26 | **<0.001**  -4.75/0.67 |
| **MOCA** | 0.768  0.36/0.275 | 0.925  0.14/0.158 | 0.904  0.12/0.692 | | 0.814  0.18/0.594 | 0.502  0.72/0.344 | 0.863  0.20/0.271 | 0.632  0.86/0.181 | 0.179  1.02/0.459 |
| **RBANS** | 0.606  8.02/0,169 | 0.94  2.10/0.304 | 0.364  15.45/0.175 | | 0.414  9.03/0.181 | 0.684  6.57/0.215 | 0.653  8.26/0.076 | 0.888  3.96/0.065 | 0.527  8.15/0.068 |
| **MFI / FSS** | **0.025**  -0.50/0.21 | 0.106  -0.64/0.18 | **0.002**  -0.74/0.35 | | **0.006**  -0.450.38 | **<0,001**  -0.86/0.58 | **0.003**  -0.74/0.35 | 0.28  -0.42/0.11 | **0.005**  -0.52/0.35 |

Data are adjusted for age and sex.

Data are analyzed at (95% CI). B =Unstandardized. R2= R-square. A p value </=0.05 is considered significant . Boldface indicates significant p-value.
